# Response to: willingness *v*. ability to pay for a universal cost-shared school food programme in Canada

**DOI:** 10.1017/S1368980024001952

**Published:** 2024-10-24

**Authors:** Suvadra Datta Gupta, Rachel Engler-Stringer

**Affiliations:** Department of Community Health & Epidemiology, University of Saskatchewan, Saskatoon, Canada

In 2019, the federal government of Canada expressed its interest in developing a school food policy for the first time. This piqued the interest of the Saskatoon Public Schools Division (SPSD), which knew they did not have the funding for free school meals for all and they requested a collaboration to understand caregivers’ attitudes towards a cost-shared school food programme (SFP). Together, we developed a survey, which, for the first time in school food literature, aimed to assess Canadian caregivers’ demand (assessed in part in terms of willingness to pay (WTP)) for a universal and cost-shared school food programme as well as other preferred attributes of such a program. This study formed the basis of the PhD thesis of the first author^(1)^. The findings of the WTP module of the study were published in Public Health Nutrition (PHN). Recently, we received comments expressing concerns about the study. This letter is in response to those concerns.

## The structure of WTP questions

Of the several methods employed to estimate consumers’ willingness to pay (WTP), contingency valuation (CV) is widely used in survey settings that ask respondents if they would like to purchase a product or service (not already available in the market) given a particular scenario^(2)^. As defined by Klose, CV is a ‘survey-based, hypothetical and direct method to determine monetary valuations’ of health/ healthcare commodities^(3)^. CV has three general techniques most often used to elicit WTP^(2,4)^. The first involves asking open-ended questions about how much the respondent is willing to pay for a ‘good or service’^(4)^. The second one is associated with using payment cards or bidding techniques. Here, the respondents pick the option closest to their individual ‘valuation’ of the product among a series of options^(4)^. The third is designing dichotomous choice models where the respondents are asked if they are willing to pay a certain amount for a given product or service pre-set by the researchers^(4)^. The double-bounded dichotomous choice (DBDC) follow-up method has been widely used and validated and is preferred by many researchers over open-ended questions or bidding techniques^(2)^ to assess WTP.

Within the CV method, how WTP questions are structured varies significantly across studies. In many studies, researchers use direct wording to inquire whether respondents are willing to pay for a hypothetical good or service. The choice between direct and indirect approaches may depend on various factors, such as the study context, objectives, respondent characteristics, the sensitivity of the topic and the researcher’s judgement. For example, König *et al.* estimated WTP in terms of giving up income by asking the maximum percentage of annual household income respondents would be ‘willing to give up’ to bear the economic costs of the measures against the coronavirus Covid-19^(5)^. By reminding respondents to think about the ’advantages and disadvantages of current treatment’, Soler and Borzykowski asked if the respondents would be ‘willing to buy’ a treatment for celiac disease^(6)^, while Jeeto *et al.* assessed WTP by comprehending respondents ‘willingness to contribute’ for pandemic preparedness^(7)^. In a study comparing locally grown products with out-of-state products, Caprio and Isengildina-Massa assessed WTP by asking, ‘What if the price of (locally) grown produce was (5 %, 10 %, 20 %, 30 %, 50 %) more expensive than out-of-state products, which one would you choose?’ and presented respondents with two answer categories – produce grown locally *v*. out-of-state produce^(8)^. In a study estimating WTP for telecare programmes among Irish respondents, Callan and O’Shea first presented the respondents with multiple proposed care programmes and asked them to rank the programmes ‘using an increase in annual taxation’^(9)^. Then, respondents were asked ‘if they would like to value the programmes through a voluntary donation as an alternative to a taxation increase’^(9)^. In a paper comparing various approaches to measure consumers’ WTP, Hofstetter *et al.* asked the respondents if the product ‘was actually offered to you for purchase in an online shop, how certain are you that you would purchase the product at the stated price’^(10)^. In another study, a dichotomous choice WTP question was structured as ‘From what you have read above, and what you may already know, do you support the proposal to build the pipe, to be funded by a once-off levy of $50 on income tax, or do you oppose it?’^(11)^. Note that all these surveys employed the CV method, described their product in a hypothetical setting and used various forms of elicitation techniques such as a bidding approach, payment cards and dichotomous choice formats with various survey objectives and contexts. There is ample scholarly discussion about how to frame WTP questions, and often researchers use indirect wordings to assess WTP to reduce bias. Therefore, we maintain that there is indeed no universally agreed-upon method to assess WTP. While a universal method could be helpful for comparing findings across studies, there are certain reasons why it is difficult to have one. WTP surveys are context-specific and are rarely replicated verbatim in other settings. Since there is no universally agreed-upon method, researchers can choose the technique that best aligns with their research interests and understanding. After reviewing various WTP elicitation and data analysis techniques, we found that many researchers prefer the DBDC technique over other elicitation methods. Therefore, we developed our WTP module based on the DBDC technique. Our study assessed WTP for a universal and cost-shared school food programme in Canada. To the best of our knowledge, no other study had assessed WTP for a Canadian SFP prior to ours. As a result, we followed the DBDC technique rather than using questions from any other survey. Therefore, the claim that we were unable to substantiate our findings by providing an example of a survey that implemented our tool prior to our study is unfair. While we do agree that our method can potentially be improved in future research, we do not believe that being the first to use it in school food research diminishes its contribution to a sparse literature.

## Willingness to pay *v*. ability to pay

The commentators are right that ability to pay (ATP) is a distinct concept from WTP. The commentators’ example, based on cigarettes, although interesting, is hypothetical, whereas the existing literature on the ability to pay provides applied examples. The amount people are willing to pay may or may not echo their ability to pay, which can be either higher or lower than their WTP^(12)^. Simply asking respondents whether they are ‘able to afford’ something does not yield actual affordability. The ability to pay refers to the total amount available to spend on goods and services (disposable income). In a study on willingness and ability to pay for health insurance, Muttaqien *et al.* measured the ability to pay by household expenditure (taken as a proxy for income) and WTP by employing a bidding game approach^(13)^. Ogundeji *et al.* calculated the ability to pay for healthcare costs by taking a 5 % expenditure-to-income ratio^(14)^. The ability to pay is a factor that determines (and sometimes acts as a constraint on) WTP^(12)^. A consumer who benefits substantially from something might be willing to pay a higher price even if their budget is limited^(12)^. On the other hand, a lower ability to pay might prevent a consumer from making a purchasing decision even if their WTP is high^(12)^. We have focused on estimating the WTP by employing a DBDC method, which numerous researchers have used to estimate WTP for goods and services – and not for assessing ability to pay. There is no need to reorient the paper saying that we measured ability to pay because DBDC technique questions are unable to measure affordability.

## Why did not we use the terms ‘willingness to pay’ directly?

The commentators raise this question ‘*why did they not simply ask “Would you be willing to pay…”’ and* ‘*Moreover, if the Saskatoon Public Schools Division was responsible for the final wording of the questions; why did the authors not simply reorient.’* When researchers use a participatory approach, they often use tools developed through meaningful involvement of their partners^(15)^. The survey iteration initially shared with SPSD asked (after presenting the hypothetical scenario), ‘Would you like to join the program for a monthly payment of ….?’. SPSD wanted to frame the question slightly differently given they did not think the word ‘join’ was appropriate for their purposes. After a few iterations of working on the questionnaire, the survey question was finally structured as ‘Would you be able to afford a daily payment of ….’. The wording of our survey was chosen carefully and cautiously after having multiple discussions with the SPSD and other school food researchers. Our study also underwent pre-testing with nine pilot tests, done in-person with participants from various socio-demographic and economic background, including both high- and low-socioeconomic status parents. In the pilot tests, we explained to our respondents that we want to measure ‘willingness to pay’ and presented them the questionnaire. We did not encounter any concerns that there was a disconnect between what we said we wanted to measure (WTP) and the wording we had in our survey (able to afford). To the degree possible in a first survey of its kind, we do believe we had content and construct validity in our survey.

We have shown our DBDC tool in multiple academic and public practitioner presentations. We included the survey instrument in Datta Gupta’s publicly available thesis and shared the instrument with anyone who was interested in replicating the technique. Multiple research groups in Canada are also currently utilising the instrument, adapting it to their local context. We have maintained full transparency in our research process and have shared our tool whenever requested. As such, we do not agree with the concern of not having transparency and reproducibility.

Lastly, there is extensive literature comparing stated preference *v*. revealed preference methods and direct *v*. indirect methods for assessing WTP. However, no method has been found to be completely reliable. Additionally, we have not come across any research that has statistically validated the use of the term ‘willing to pay’ as necessary for a tool measuring WTP. Even though our study was not intended to, and also not able to, validate the various data structures of the WTP module, the response letter states the commentators’ views on the module rather than on the scholarly discussion of the various WTP techniques. Considering that a multitude of approaches are used to measure WTP (as detailed above), and that estimating ATP varies significantly from the WTP estimation techniques, this challenges the commentators’ concern that our study was methodologically incorrect because the commentators’ preferred terms were not included.

To conclude, we want to thank the commentators for spurring the further development of our understanding of the subtleties of these types of survey questions and the scholarly debate we have been engaged in. Research that responds to real-world questions is messy and complex, but critical for understanding our complex world and the interventions we make in it to improve population health. Most importantly, we hope that the original publication and these letters will invite others to conduct similar research in Canadian and other contexts and learn from this debate to further strengthen the school food literature, where significant gaps exist.
